# Correction of anterior open bite of varying severity using clear aligner therapy—A case series

**DOI:** 10.1002/ccr3.6277

**Published:** 2022-08-29

**Authors:** Waddah Sabouni, Adith Venugopal, Samar M. Adel, Nikhilesh Vaid

**Affiliations:** ^1^ Private Practice Invislign Centre Dubai United Arab Emirates; ^2^ Department of Orthodontics Saveetha Dental College, Saveetha Institute of Medical and Technical Sciences, Saveetha University Chennai India; ^3^ Department of Orthodontics University of Puthisastra Phnom Penh Cambodia; ^4^ Faculty of Dentistry Alexandria University Alexandria Egypt

**Keywords:** anterior open bite, CAT, clear aligners, incisor extrusion, posterior intrusion, TAD

## Abstract

In open bite cases, a comprehensive diagnostic differentiation is crucial in determining the best corrective therapy. In non‐surgical open bite treatment, fixed appliances, either labial or lingual, are usually employed. With the addition of extra‐radicular screws, more sophisticated orthodontic movements may now be performed without the necessity for orthognathic surgery. Clear aligner therapy, on the contrary, has grown in popularity as a treatment option for more complex cases, such as open bite malocclusions. This article discusses three cases with an anterior open bite that were treated using various mechanics as dictated by the malocclusion. Case 1 was addressed wholly using clear aligner therapy, with careful consideration of attachment geometry and mechanics. Case 2 with clear aligner therapy, attachment geometry selection, and vertical elastics; and Case 3 with clear aligner therapy, attachments, and temporary anchorage devices.

## 1 INTRODUCTION

The etiology of an anterior open bite (AOB) is multifactorial in nature. Unfavorable growth patterns, oral habits, respiratory factors, and neuromuscular imbalances have been suggested to play a role. AOB results in significant esthetic and functional concerns often, including difficulties with breathing, chewing, and speaking.^1^ The treatment outcome should improve both: esthetic and function. Finally resulting in satisfaction; as evaluated in national dental practice‐based research from the United States.^2^

The scope of Clear Aligner Therapy (CAT) has greatly increased over the past decade or so from treating merely a mild to moderate crowding to a well‐controlled sophisticated therapeutic solution for complex malocclusions too. Though scholarly evidence for the system is still in infancy,^3^, ^4^ published case reports have showcased extremely encouraging outcomes with complex cases.^5^, ^6^ These cases report novelties in the literature such as those involving extractions, open bites, cross bites, and class II malocclusions. The fact that patients undergoing CAT demonstrate better quality of life (QoL) scores during treatment helps the practitioner to imbibe such treatment for their patients—also to tackle challenging cases.^7^, ^8^

Recent clinical literature has demonstrated how an AOB can be efficiently addressed with posterior intrusion accompanied by retraction of incisors using CAT, both with and without adjuncts.^9^

This article describes three cases with an AOB, which have been treated with different mechanics as mandated by the malocclusion. Case 1 was treated with CAT entirely with judicious use of attachment geometry and mechanics, Case 2 was treated with CAT, attachment geometry selection and vertical elastics, and finally Case 3 was treated with CAT, attachments, and temporary anchorage devices.

## 2 CASE REPORT 1

An 18‐year‐old male patient complained of a gap between his upper and lower teeth and associated stigmatism. Extra‐oral examination showed a symmetrical appearance with reduced upper incisal display while smiling. Intra‐oral examination revealed an AOB, class I molar relationship on both sides, class I canine relation on the right side, and an end on class II canine relationship on the left side with mild crowding and rotations on the lower anterior segment. (Figure 1).

**FIGURE 1:**
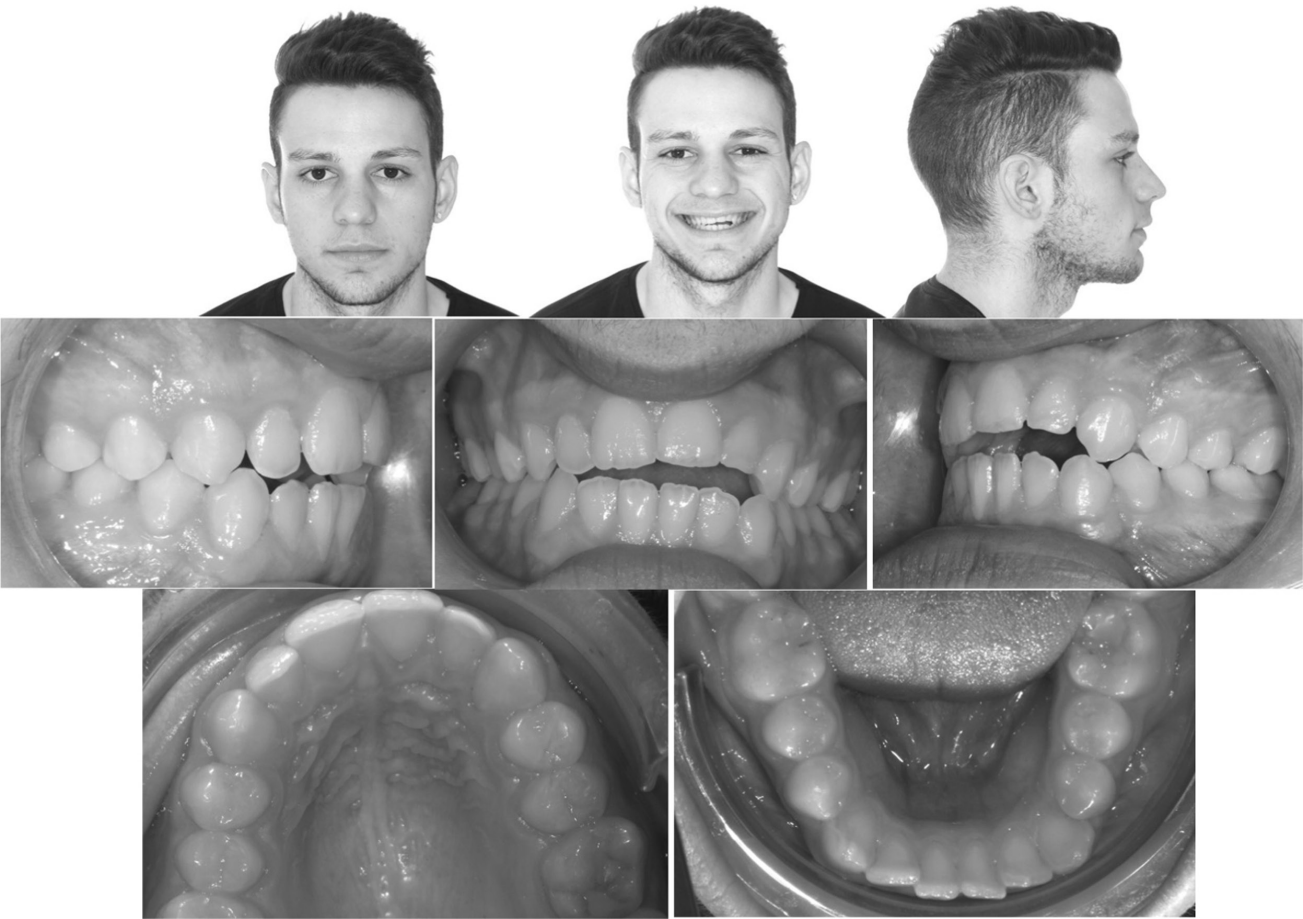
Case 1: Pre‐treatment intra‐oral and extra‐oral pictures

Radiographic evaluation showed proclined upper/lower incisors (pre IMPA: 99°; pre U1SN: 115°), a skeletal class I base‐relationship (pre ANB: 3.6°) with normo‐divergent mandibular plane angle (pre FMA: 26°). (Figure 2; Table 1).

**FIGURE 2:**
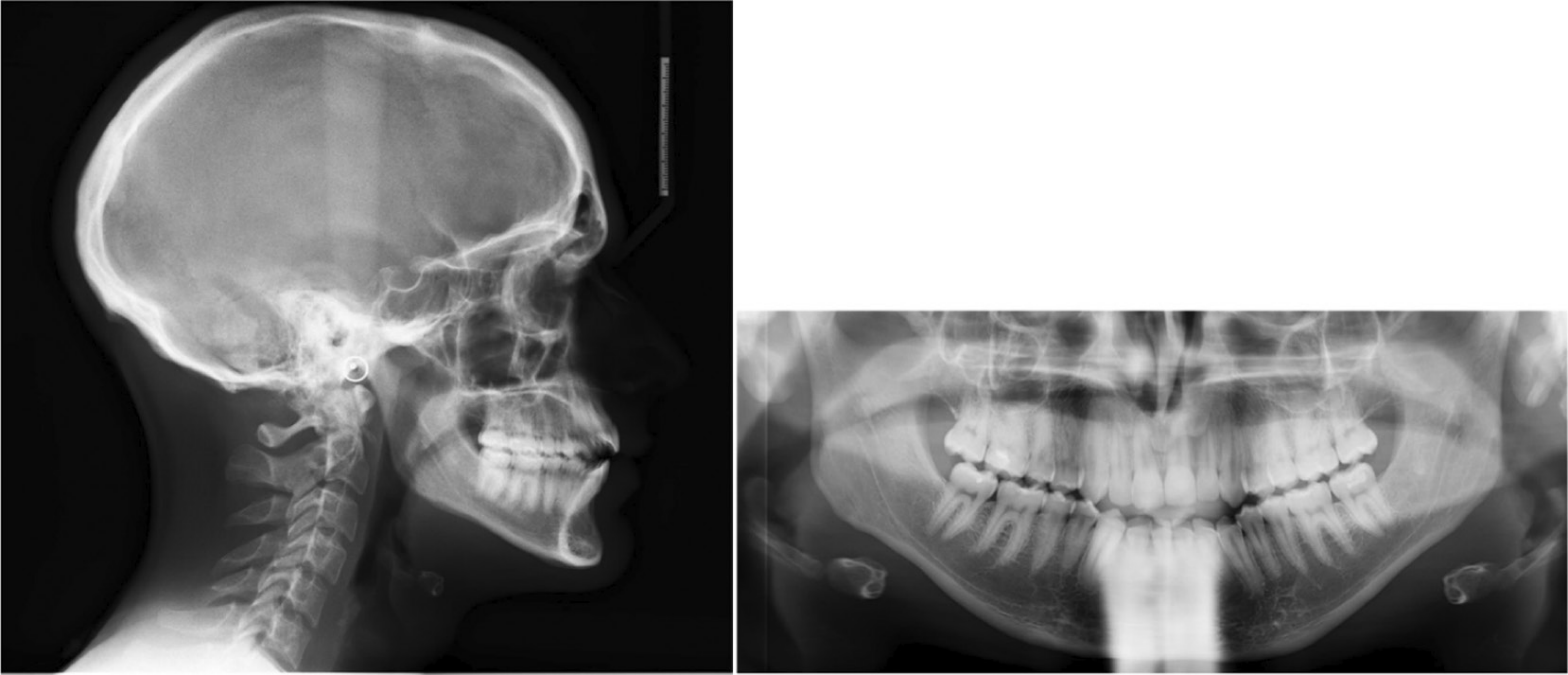
Pre‐treatment radiographs

**TABLE 1:** Cephalometric analysis—Case 1

Variable	Mean	Pre‐treatment	Post‐treatment
SNA (dg)	82 ± 3	86.75	86.16
SNB (dg)	79 ± 3	83.17	82.62
ANB (dg)	3 ± 1	3.58	3.54
IMPA (dg)	92 ± 5	99.15	92.62
U1‐SN (dg)	102 ± 6	115.06	104.65
FMA (dg)	26 ± 3	26.30	26.85

Conventional gingival beveled attachments were placed on the labial and lingual surfaces of the dentition (upper labial attachments: #14, #12, #11, #21, #22, #23, #24; upper palatal attachments: #13, #12, #11, #21, #22; lower labial attachments: #34, #33, #32, #31, #41, #42, #43). (Figure 3) The objectives were to extrude the maxillary anterior teeth and marginally intrude the maxillary posterior teeth in order to gain closure of the bite. This would increase the maxillary incisal show resulting in a more pleasing smile line. (Figure 4) Forty‐eight upper and lower aligners were used to close the open bite, de‐rotate and alleviate the crowding on the lower anterior teeth using various orthodontic tooth movements.

**FIGURE 3:**
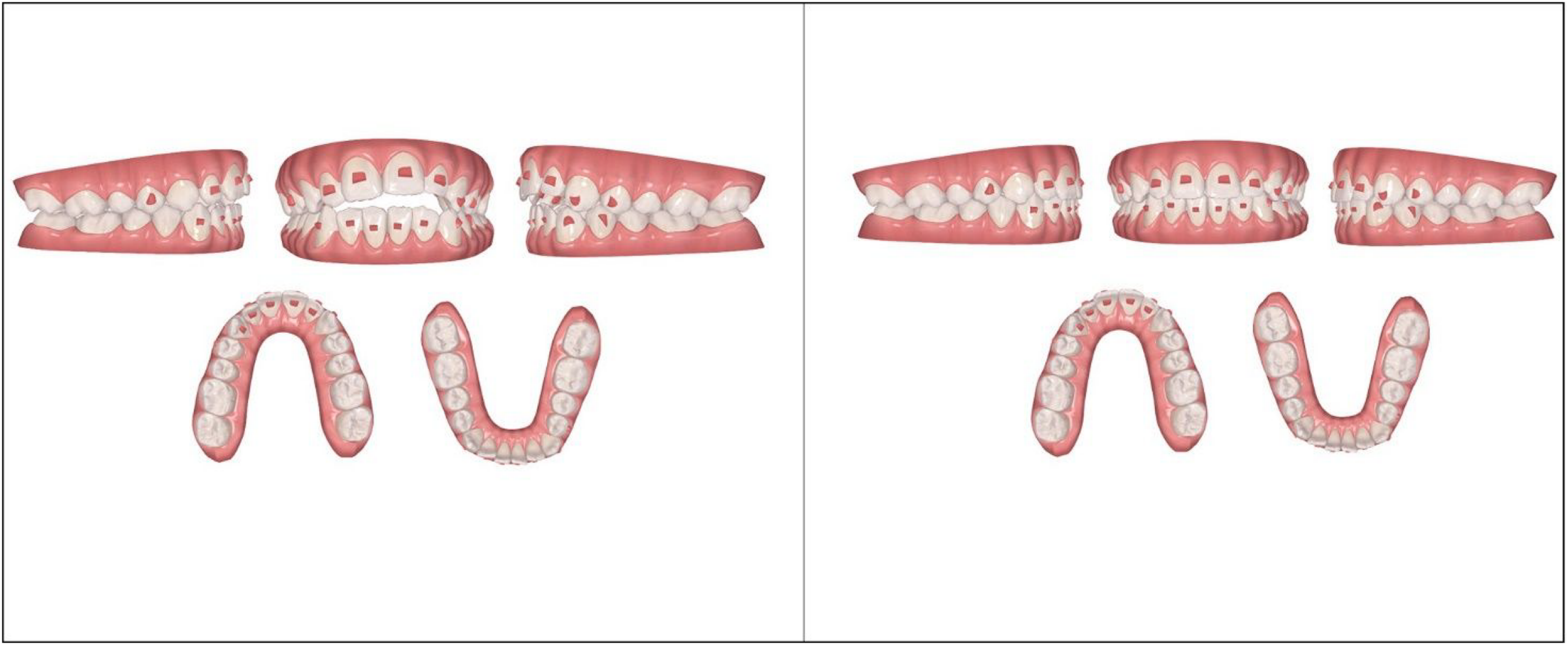
Pre‐ and post‐treatment ClinCheck™ assessment

**FIGURE 4:**
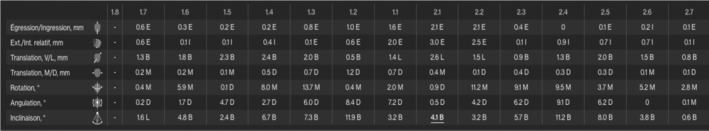
Planned tooth movement on the ClinCheck™ software

The velocity of tooth movement per aligner was set at 0.125 mm in order to deliver minimal forces. Since the patient demonstrated reduced upper incisal display, extrusion was planned for the upper incisal area (#12: 0.6 mm; #11: 2 mm; #21: 3 mm; #22: 2.5 mm).

By the end of the fortieth aligner, an edge‐to‐edge bite was noted, demonstrating proclination of lower anterior teeth. To avoid additional refinements in order to correct the overjet, the clinician placed punch hooks on the lower canine region of the subsequent aligners to attach class III intermaxillary elastics (3/16, 3.5 Oz)—to a lingual button on the upper first molars. (Figure 5).

**FIGURE 5:**
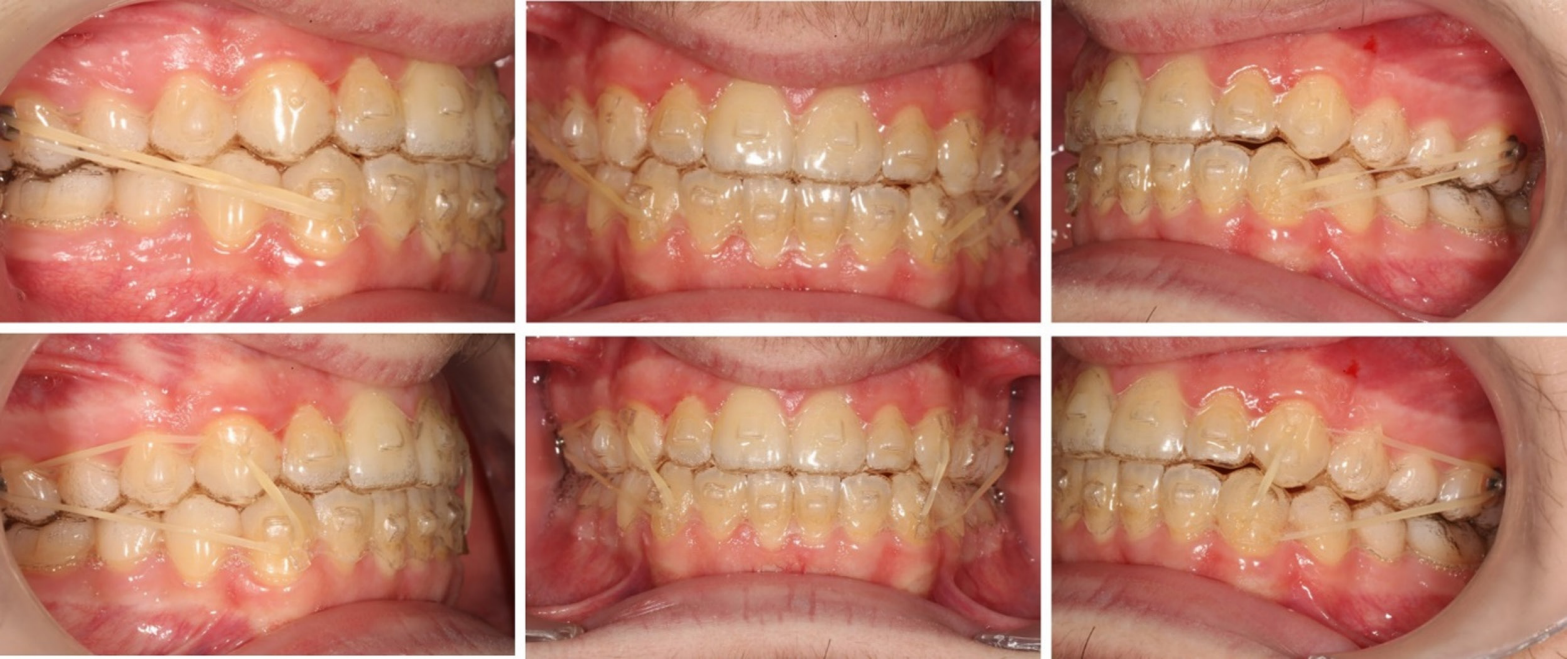
Mid‐treatment pictures showing bite closure

After 12 months of aligner treatment, the AOB was closed, the rotations and crowding on the lower incisal region were corrected and a good canine guidance had been achieved bilaterally, with a physiological overjet, overbite, and normal inclinations of upper and lower anterior teeth. (Figure 6; Figure 7; Table 1).

**FIGURE 6:**
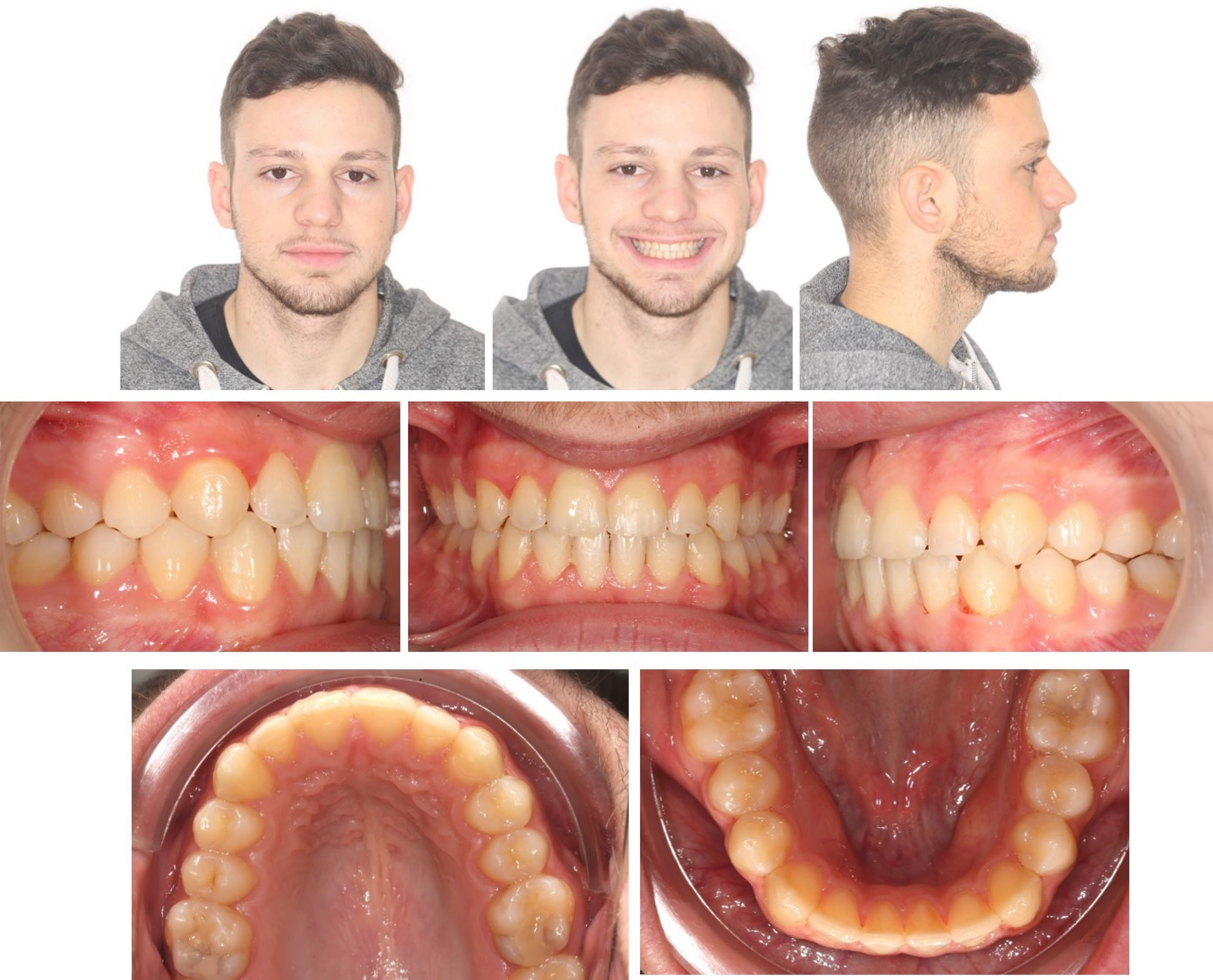
Post‐treatment intra‐oral and extra‐oral pictures

**FIGURE 7:**
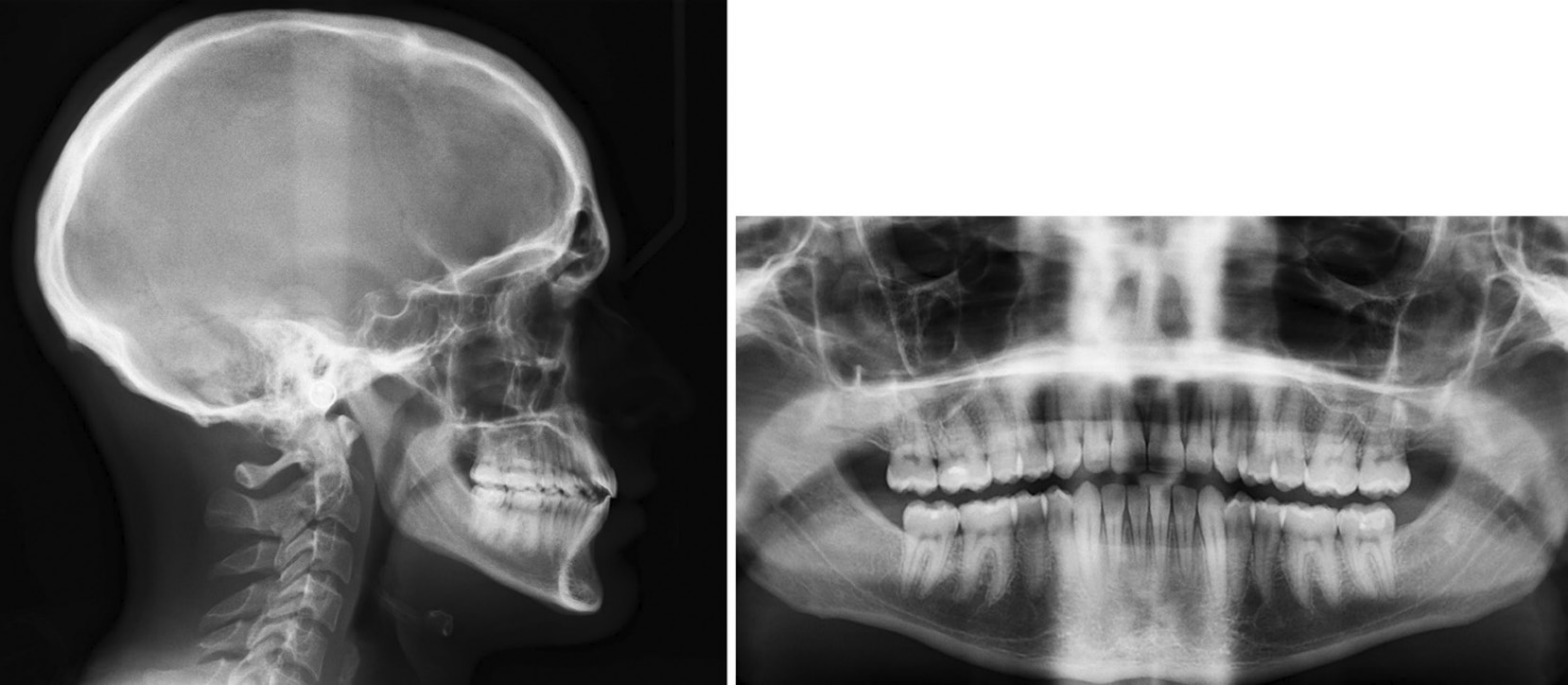
Post‐treatment radiographs

The final ClinCheck™ projections closely matched the post treatment results; frontal superimpositions of the ClinCheck™ pre‐treatment analysis and post‐treatment projection indicated the amount of extrusion needed for closure of the AOB. (Figure 8).

**FIGURE 8:**
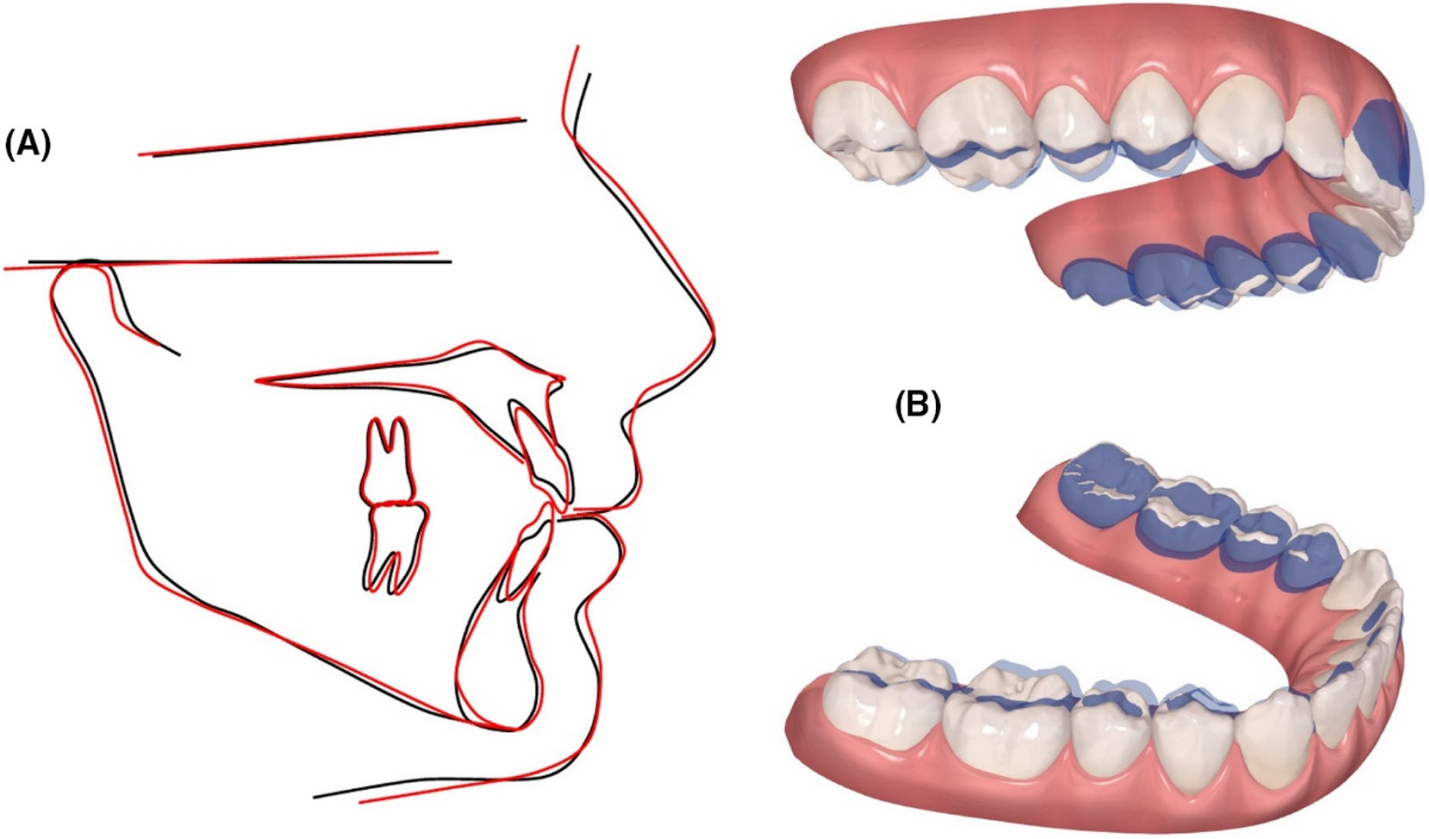
(A) Pre‐ and post‐treatment cephalometric tracings superimposed on the SN plane at S: showing no movement of the posterior dentition and extrusion of the anterior dentition. (B) Dental changes as seen in the ClinCheck™ software

Following the treatment, a fixed lingual retainer was placed on the lower arch in addition to the Vivera™ retainers on the upper and lower arches to prevent further posterior extrusion and facilitate minimal anterior extrusion in order to overcorrect the overbite. Postretention pictures after a year showed minimal tooth movement and well aligned teeth. (Figure 9).

**FIGURE 9:**
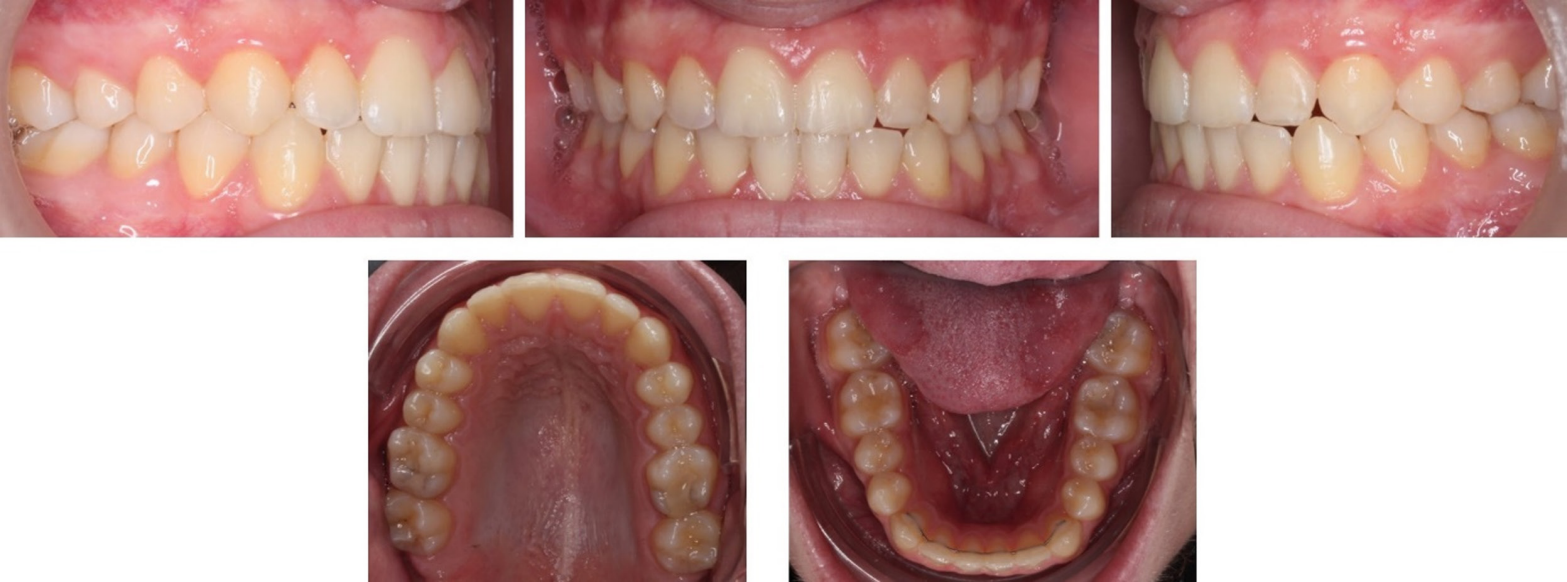
One‐year postretention intra‐oral pictures

## 3 CASE REPORT 2

A 25 year old female patient complained of a gap between her front teeth and the inability to chew food properly. Extraoral examination showed a symmetrical appearance with reduced upper incisal display smiling. Intra‐oral examination revealed an AOB with a class I molar relationship on both sides, half a cusp class II canine relation on the right side and a projected end on class II canine relationship on the left side with mild crowding and rotations on the upper and lower anterior segments. (Figure 10).

**FIGURE 10:**
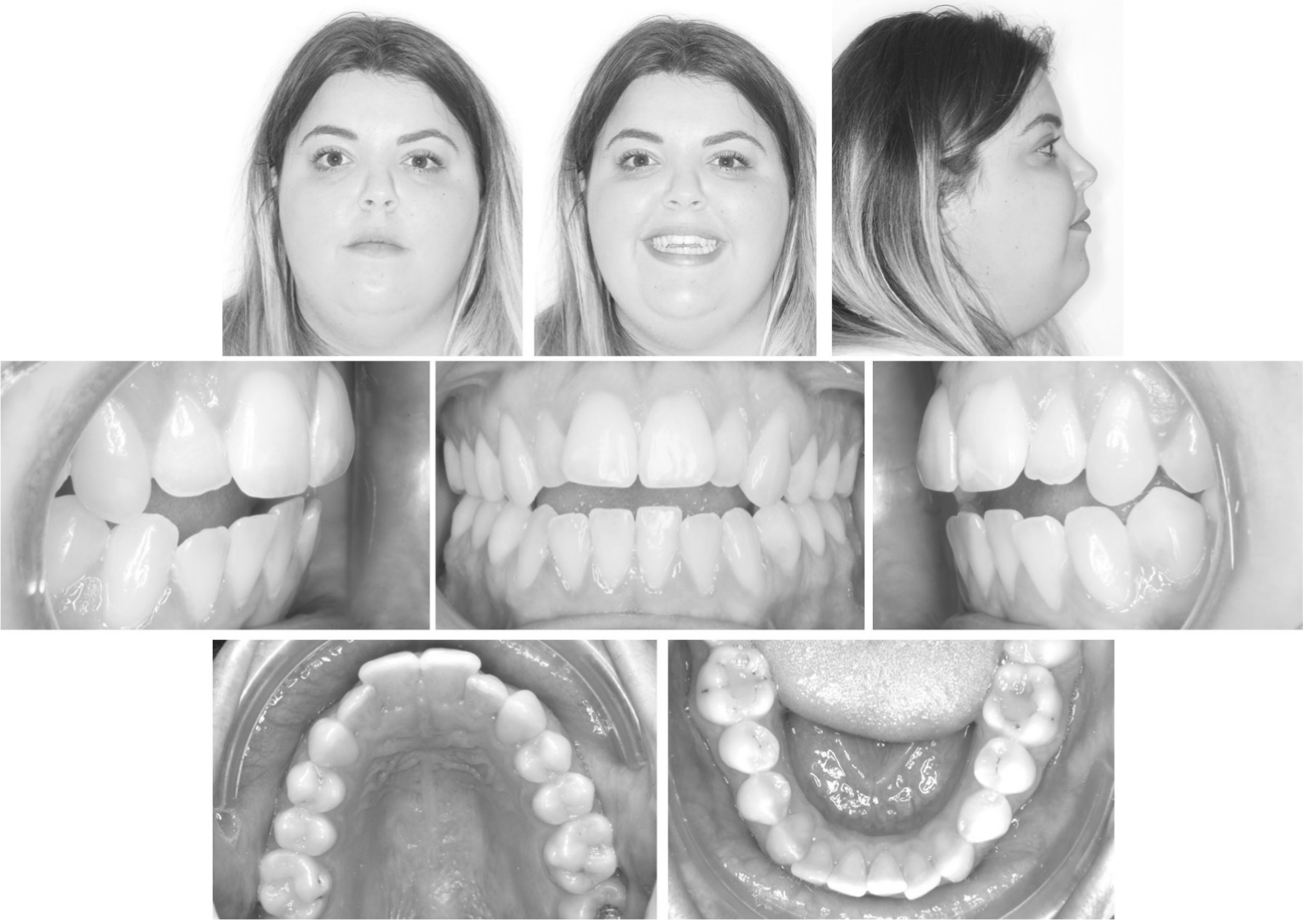
Case 2—Pre‐treatment intra‐oral and extra‐oral pictures

Radiographic evaluation showed proclined upper and lower incisors (pre IMPA: 97°; pre U1SN: 113°) on a skeletal class II base relationship (pre ANB: 5.7°) with normo‐divergent mandibular plane angle (pre FMA: 25°). (Figure 11; Table 2).

**FIGURE 11:**
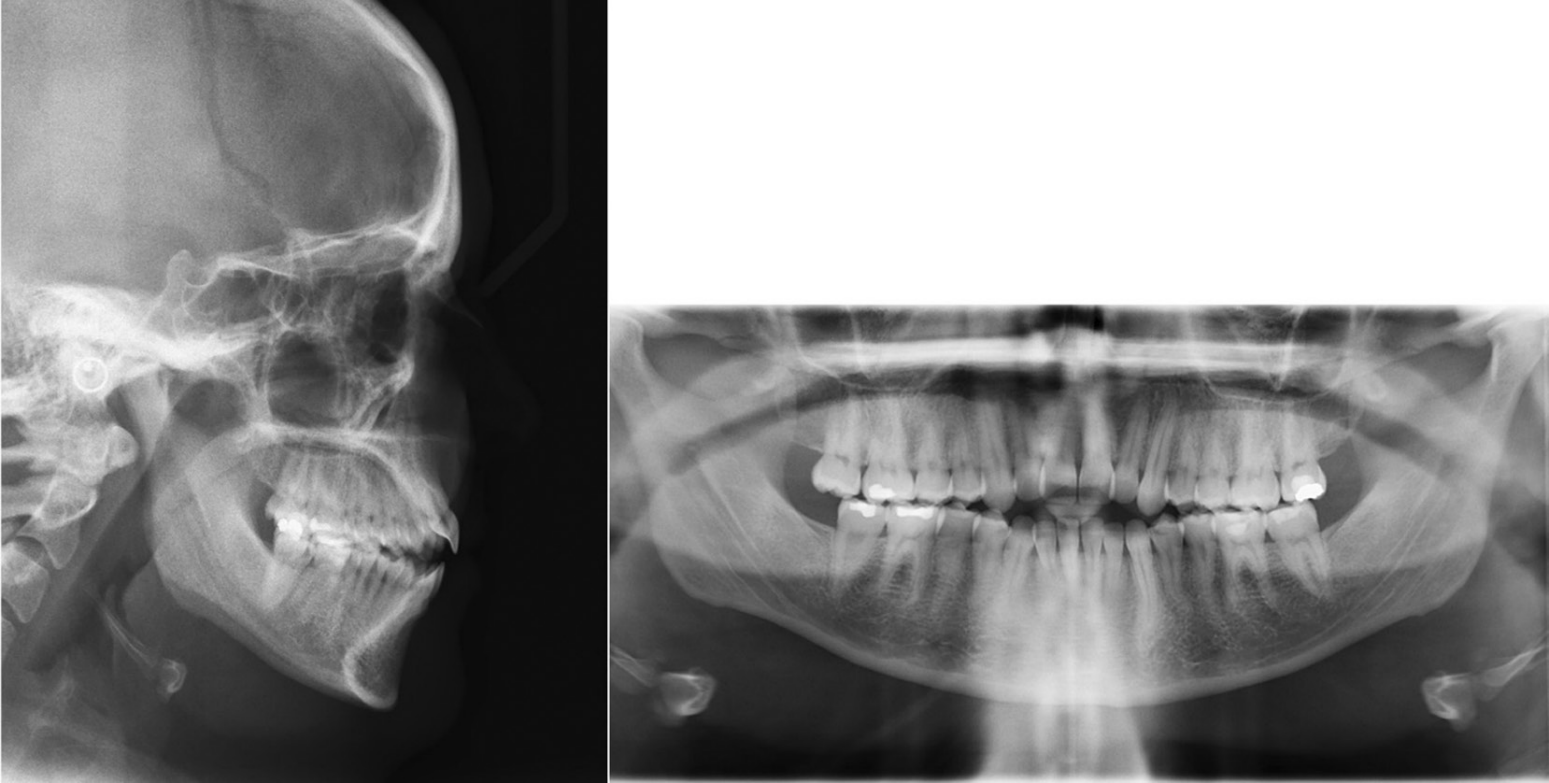
Pre‐treatment radiographs

**TABLE 2:** Cephalometric analysis—Case 2

Variable	Mean	Pre‐treatment	Post‐treatment
SNA (dg)	82 ± 3	85.85	86.05
SNB (dg)	79 ± 3	80.17	81.62
ANB (dg)	3 ± 1	5.68	4.43
IMPA (dg)	92 ± 5	96.79	91.61
U1‐SN (dg)	102 ± 6	112.89	104.57
FMA (dg)	26 ± 3	25.06	25.63

Conventional beveled attachments were placed on the labial and lingual surfaces of the dentition (upper labial attachments: #15, #14, #13, #12, #11, #21, #22, #23, #24, #25; upper palatal attachments: #13, #12, #11, #21, #22, #23; lower labial attachments: #34, #33, #32, #31, #42, #43, #44, #45; lower lingual attachments: #31, #41).

Seventeen upper and lower aligners were used to initially extrude the upper anterior segments (at the rate of 2 weeks per aligner) by approximately 3 mm and also to intrude the upper posterior segment by 1 mm.(Figure 12, Figure 13) In order to achieve this, the patient was asked to wear additional inter maxillary elastics (1/8, 3.5 Oz) from the hooks fabricated on the upper canine to the ones on the lower canine and premolar, in a triangular fashion and only at nighttime. (Figure 14).

**FIGURE 12:**
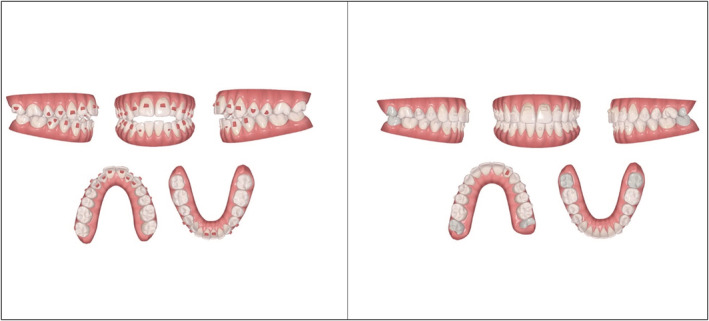
Pre‐ and post‐treatment ClinCheck™ assessment

**FIGURE 13:**
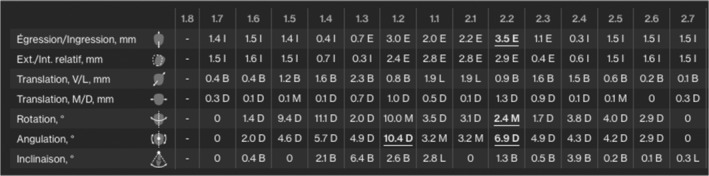
Planned tooth movement on the ClinCheck™ software

**FIGURE 14:**
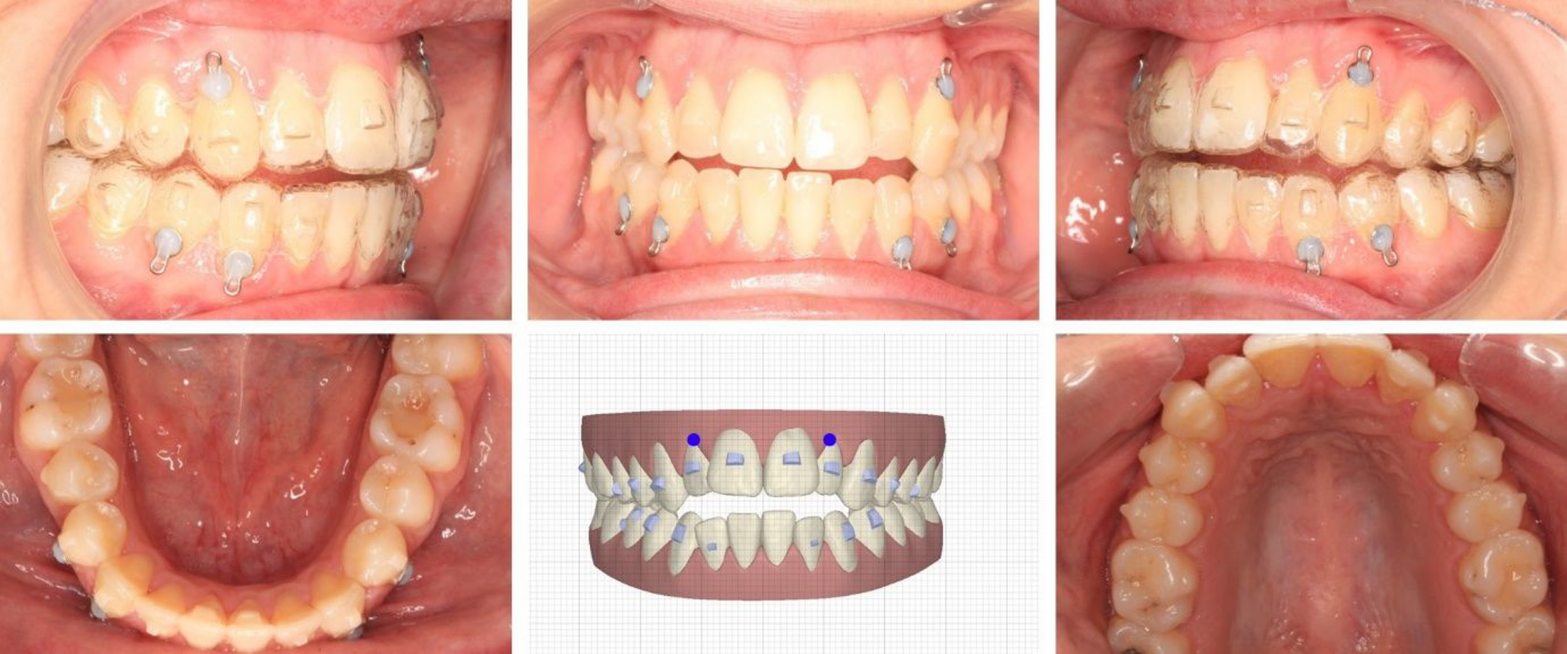
Mid‐treatment pictures

The first refinement consisting of nine sets of aligners were ordered, since the bite closure on the lateral incisor was inadequate. Bootstrap mechanics was applied to the upper lateral incisors using palatally placed lingual buttons and elastics running to the cleat on the aligners in order to extrude them reliably. (Figure 15) A final refinement consisting of five sets of aligners were ordered to refine the inclinations and angulations of all the teeth and settle the occlusion in the best possible way. Aligners during the refinement stages were worn at the rate of one aligner/week.

**FIGURE 15:**
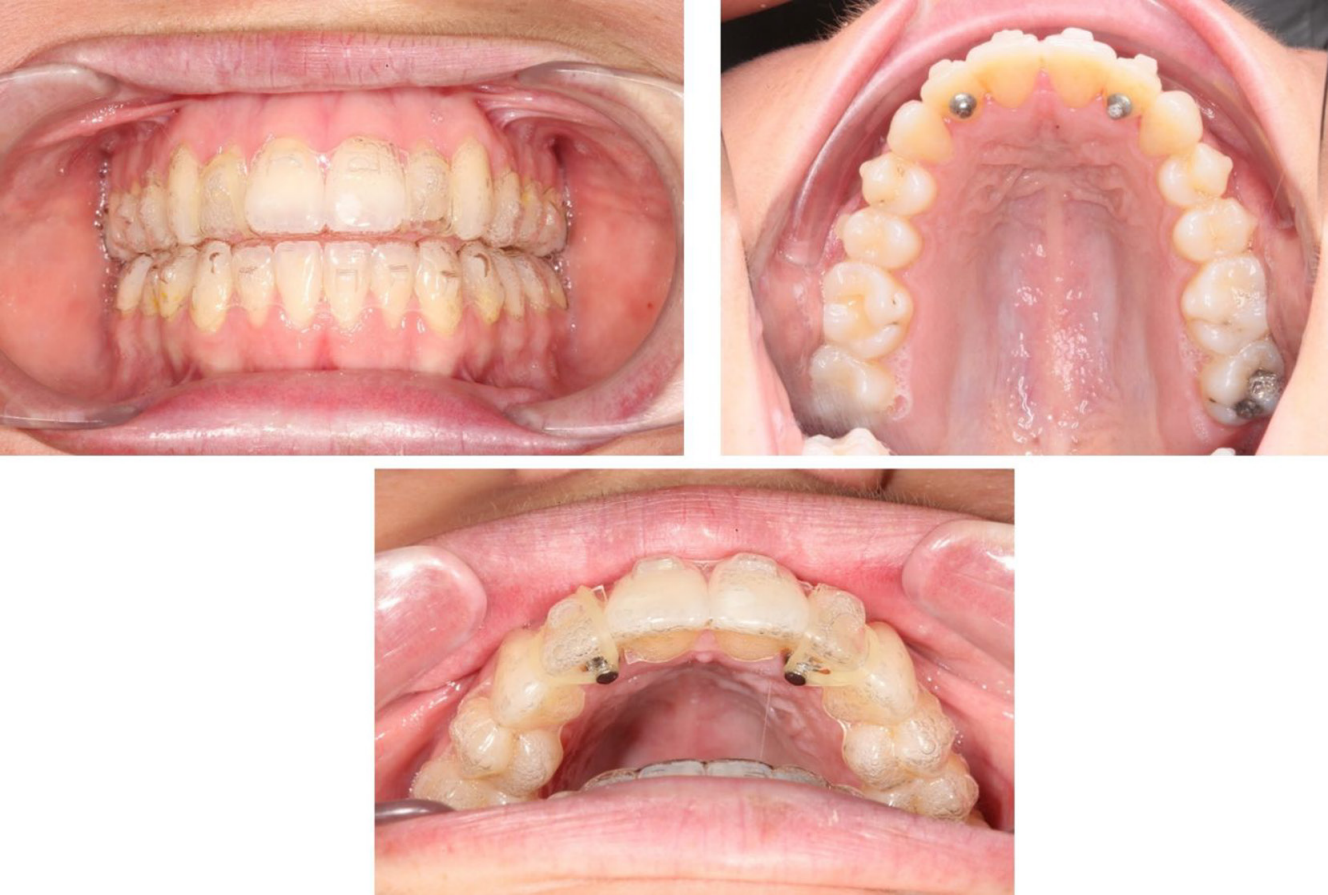
Bootstrap mechanics for lateral incisor extrusion

The AOB had closed, the rotations and crowding on the social sixes had been addressed, and canine guidance had been obtained bilaterally, with a functional overjet and overbite, after 35 months of aligner treatment. (Figure 16; Figure 17).

**FIGURE 16:**
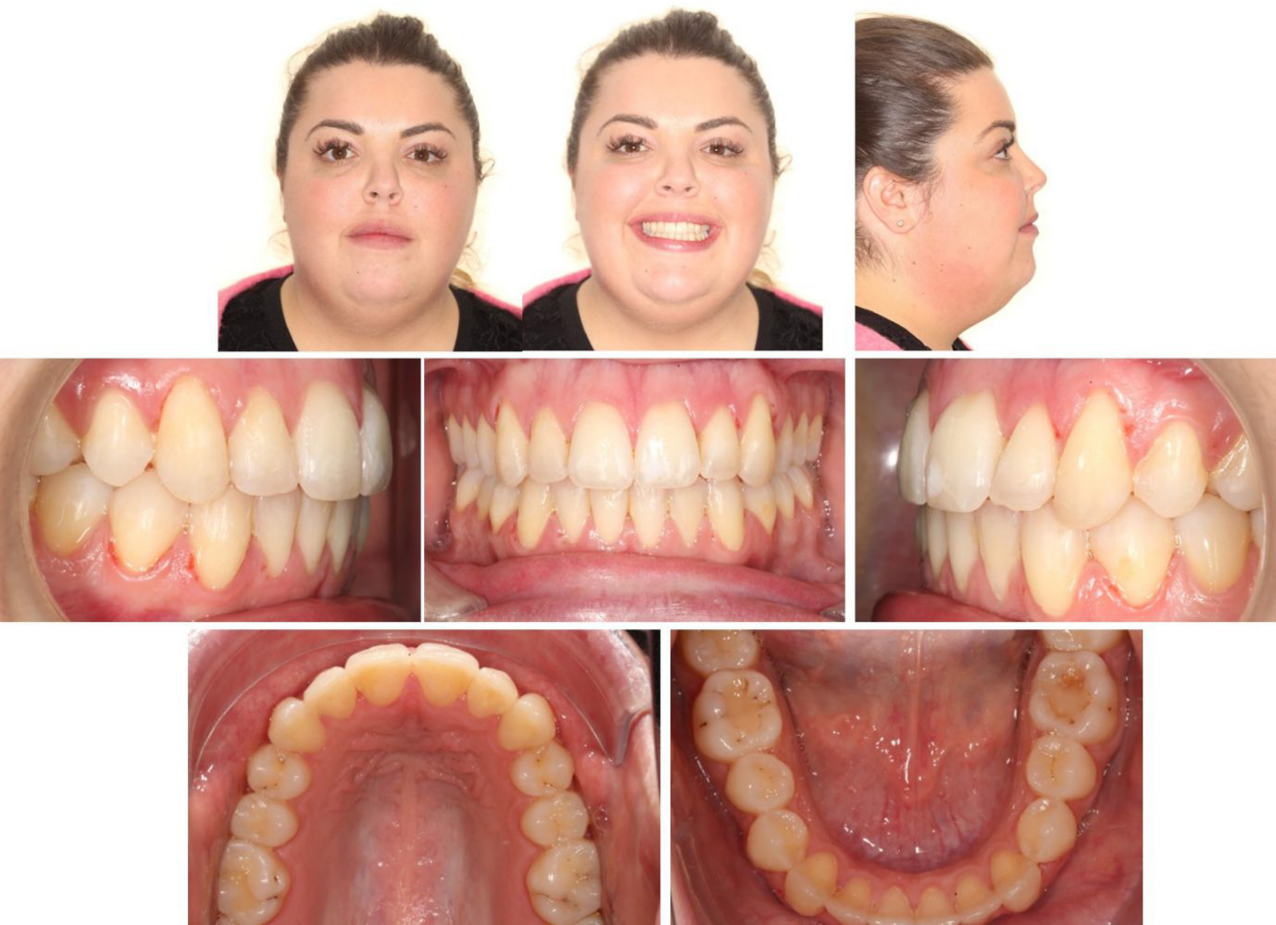
Post‐treatment intra‐oral and extra‐oral pictures

**FIGURE 17:**
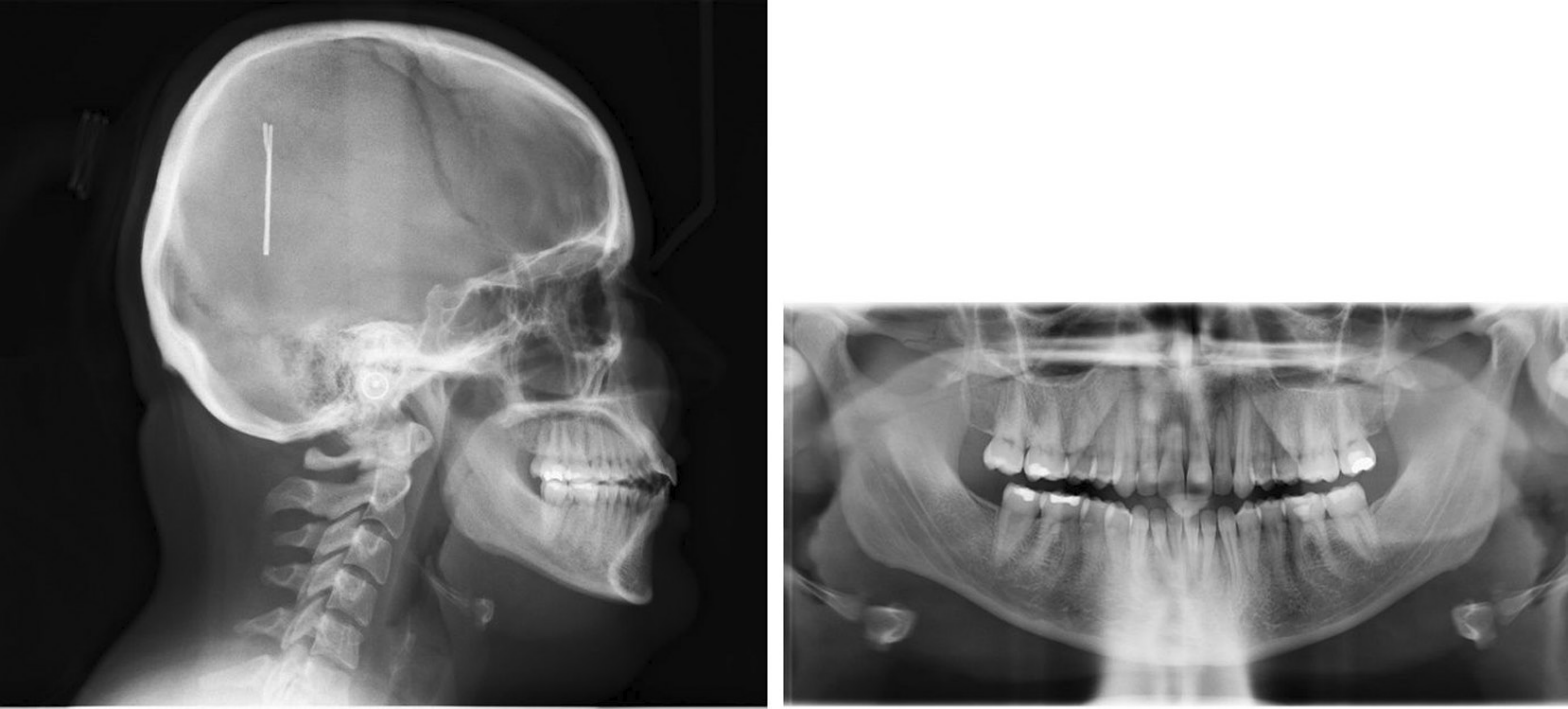
Post‐treatment radiographs

The final ClinCheckTM projections were nearly identical to the post‐treatment results; frontal superimpositions of the ClinCheckTM pretreatment analysis and post‐treatment projection revealed the amount of anterior extrusion and molar intrusion required to close the open bite. (Figure 18).

**FIGURE 18:**
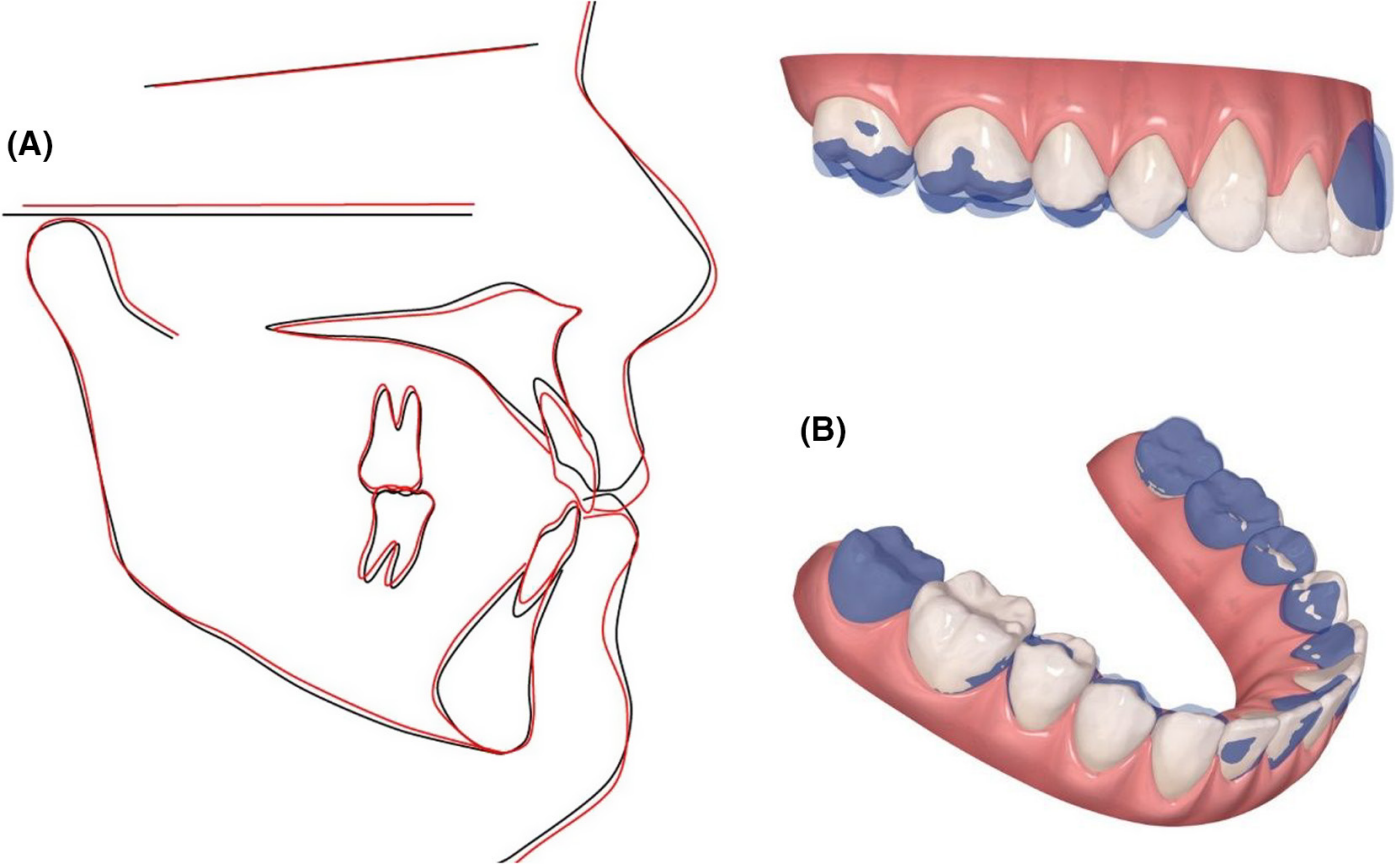
(A) Pre‐ and post‐treatment cephalometric tracings superimposed on the SN plane at S: showing no movement of the posterior dentition and extrusion of the anterior dentition. (B) Dental changes as seen in the ClinCheck™ software

Following the treatments, Vivera™ retainers on the upper and lower arches, to restrict further posterior extrusion and to retain the final result were provided. One‐year post‐retention pictures show further deepening of the bit, better occlusal outcomes, and well aligned arches. (Figure 19).

**FIGURE 19:**
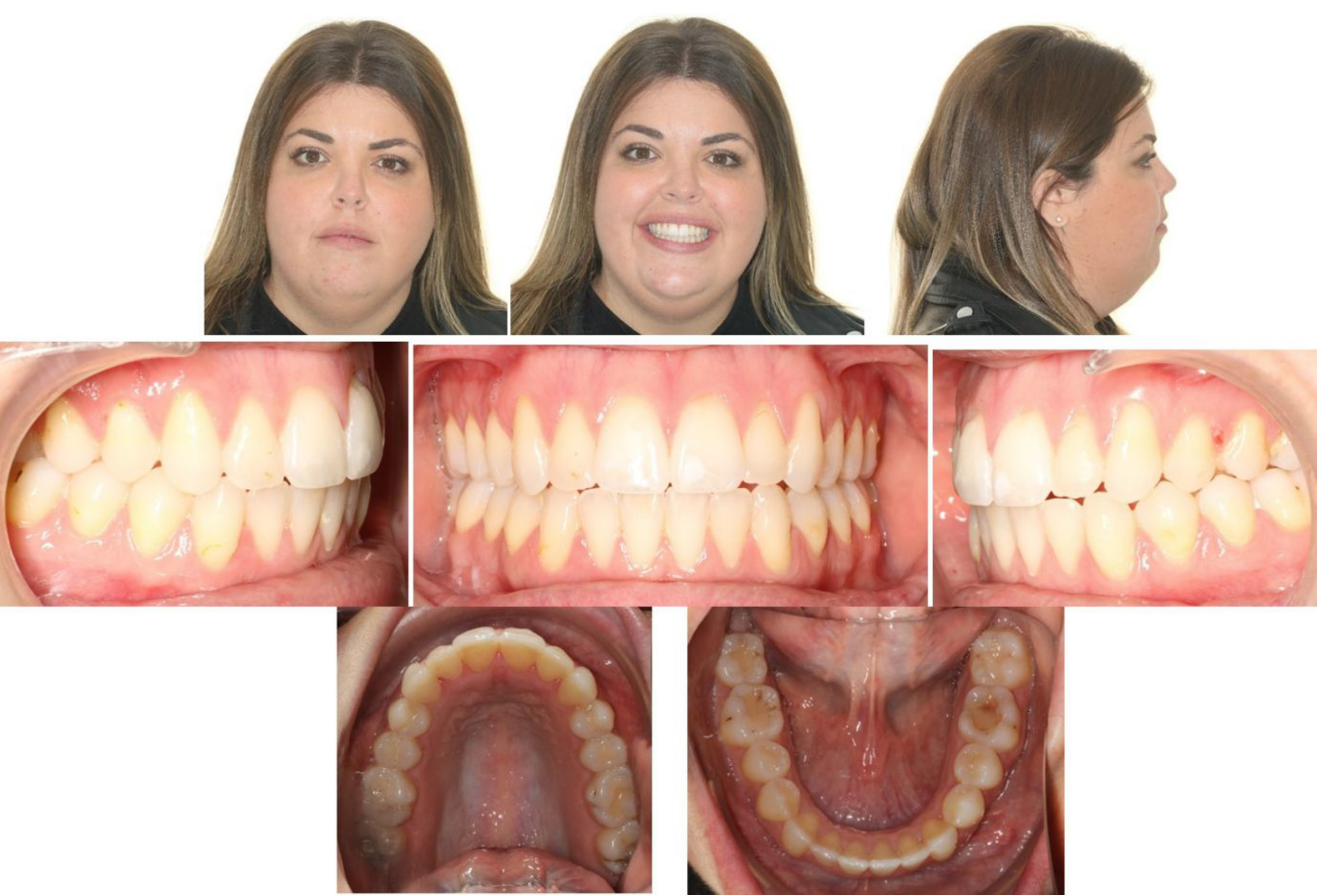
One‐year extra‐oral and intra‐oral post retention pictures

## 4 CASE REPORT 3

A 22‐year‐old female patient presented with a history of amelogenisis imperfecta and prior orthodontic treatment.

Her chief complaint was that she was unable to chew and there was a gap between her upper and lower teeth because of which she could not smile confidently. Extraoral examination showed a symmetrical appearance with a convex profile and adequate upper incisal display on smiling. Intra‐oral examination revealed an AOB, no contact on the posteriors (up to the region of the second molars) with a projected class II molar and canine relationship on both sides; an overjet of 8 mm. (Figure 20).

**FIGURE 20:**
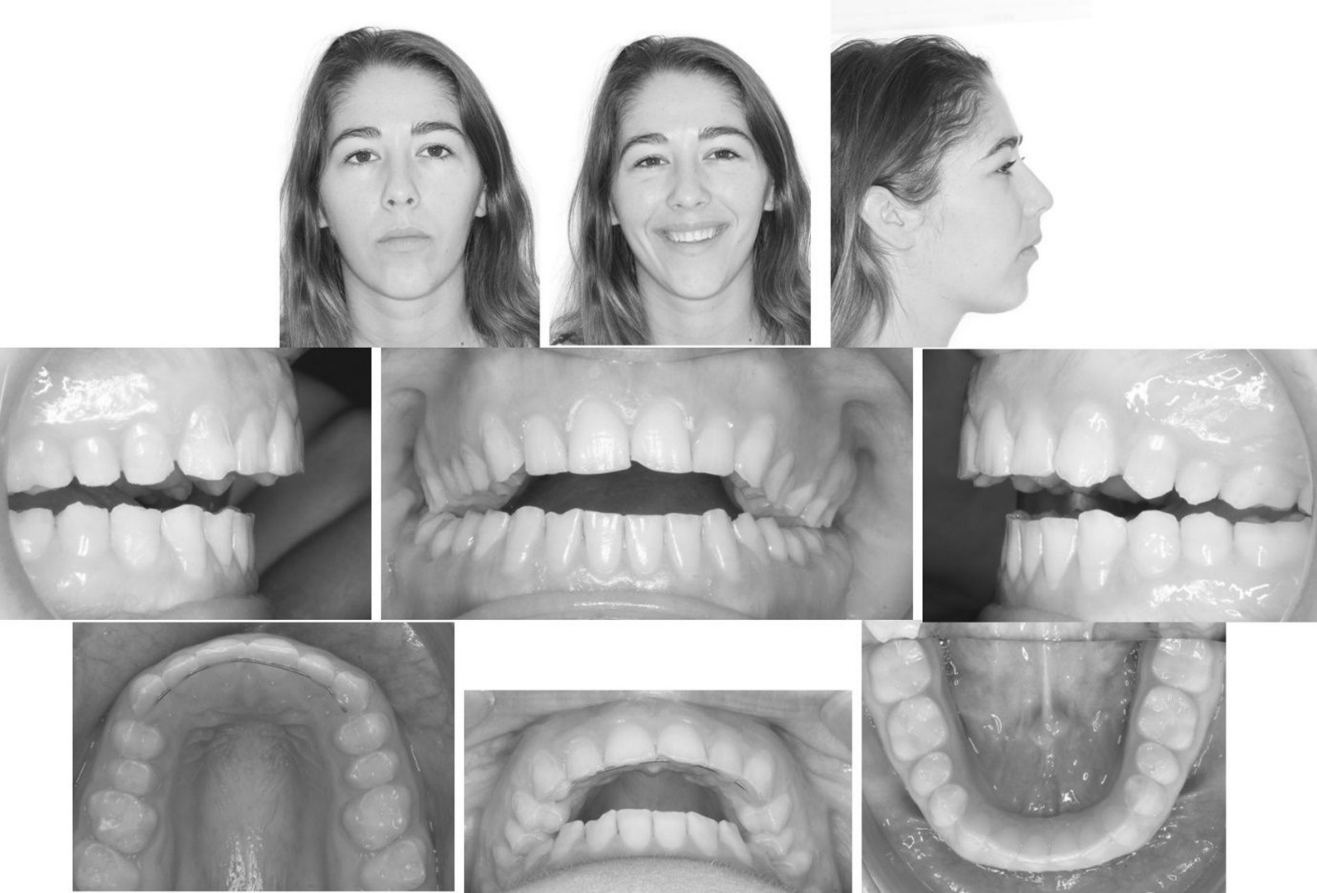
Case 2—Pre‐treatment intra‐oral and extra‐oral pictures

Radiographic evaluation showed upright incisors (pre IMPA: 92°; pre U1SN: 99°) on a skeletal class II base relationship (pre ANB: 7°) with a slightly steep mandibular plane angle (pre FMA: 28.83°). (Figure 21; Table 3).

**FIGURE 21:**
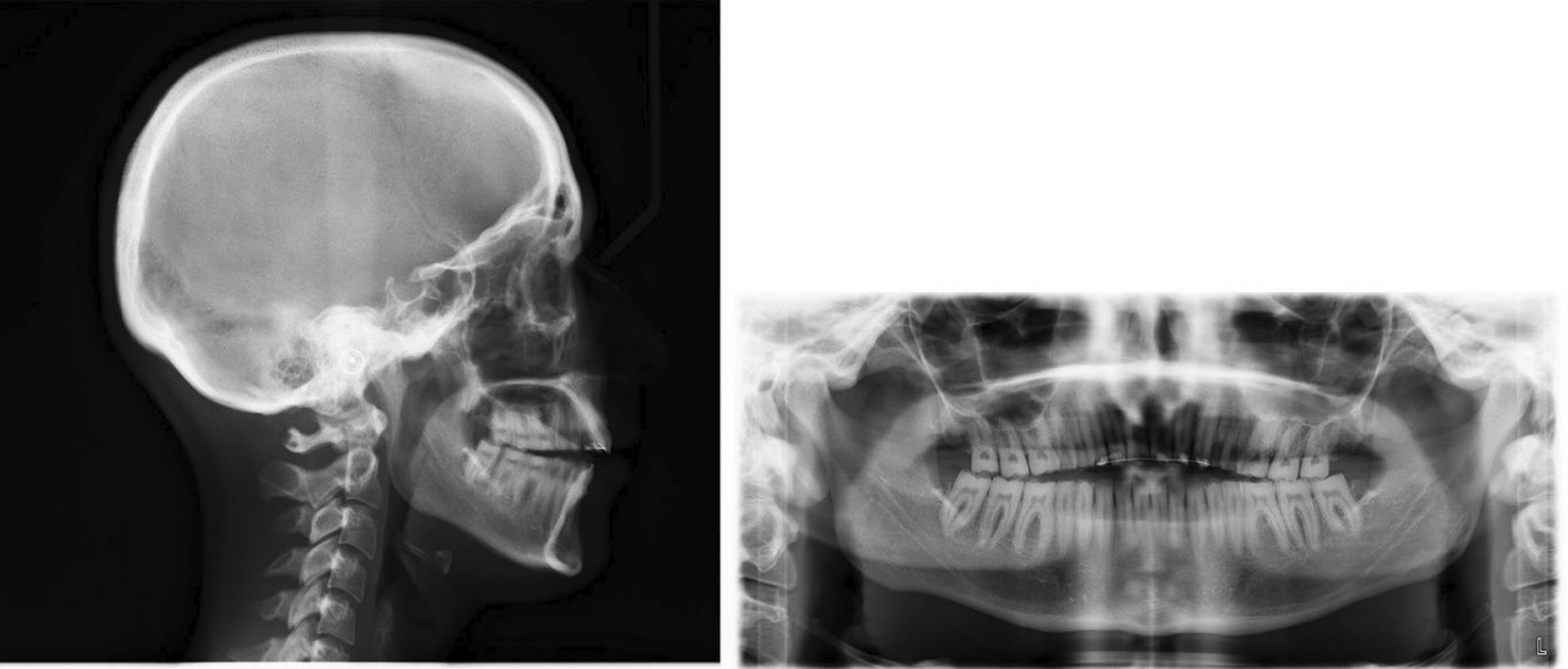
Pre‐treatment radiographs

**TABLE 3:** Cephalometric analysis—Case 3

Variable	Mean	Pre‐treatment	Post‐treatment
SNA (dg)	82 ± 3	80.80	79.27
SNB (dg)	79 ± 3	73.62	74.79
ANB (dg)	3 ± 1	7.18	4.48
IMPA (dg)	92 ± 5	92.49	94.75
U1‐SN (dg)	102 ± 6	98.74	95.60
FMA (dg)	26 ± 3	28.83	26.15

Since the patient suffered from amelogenesis imperfecta, a potential re‐treatment with braces was not an option. CAT with 20 sets of aligners were planned using dual attachments on the labial and lingual surfaces to intrude the maxillary posterior teeth and marginally extrude the maxillary anterior teeth. (Figure 22).

**FIGURE 22:**
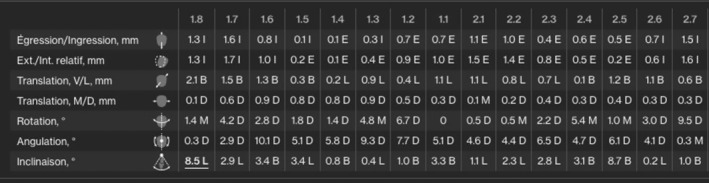
Planned tooth movement on the ClinCheck™ software

The conventional attachments were also placed on the labial and lingual surfaces (upper labial attachments: #17, #15, #12, #11, #21, #22, #25, #27; upper palatal attachments: #13, #12, #11, #21, #22, #23; lower labial attachments: #37, #35, #33, #32, #42, #43, #45, #47).

Optimized attachments were placed on the labial surfaces of #31 and #41. (Figure 23).

**FIGURE 23:**
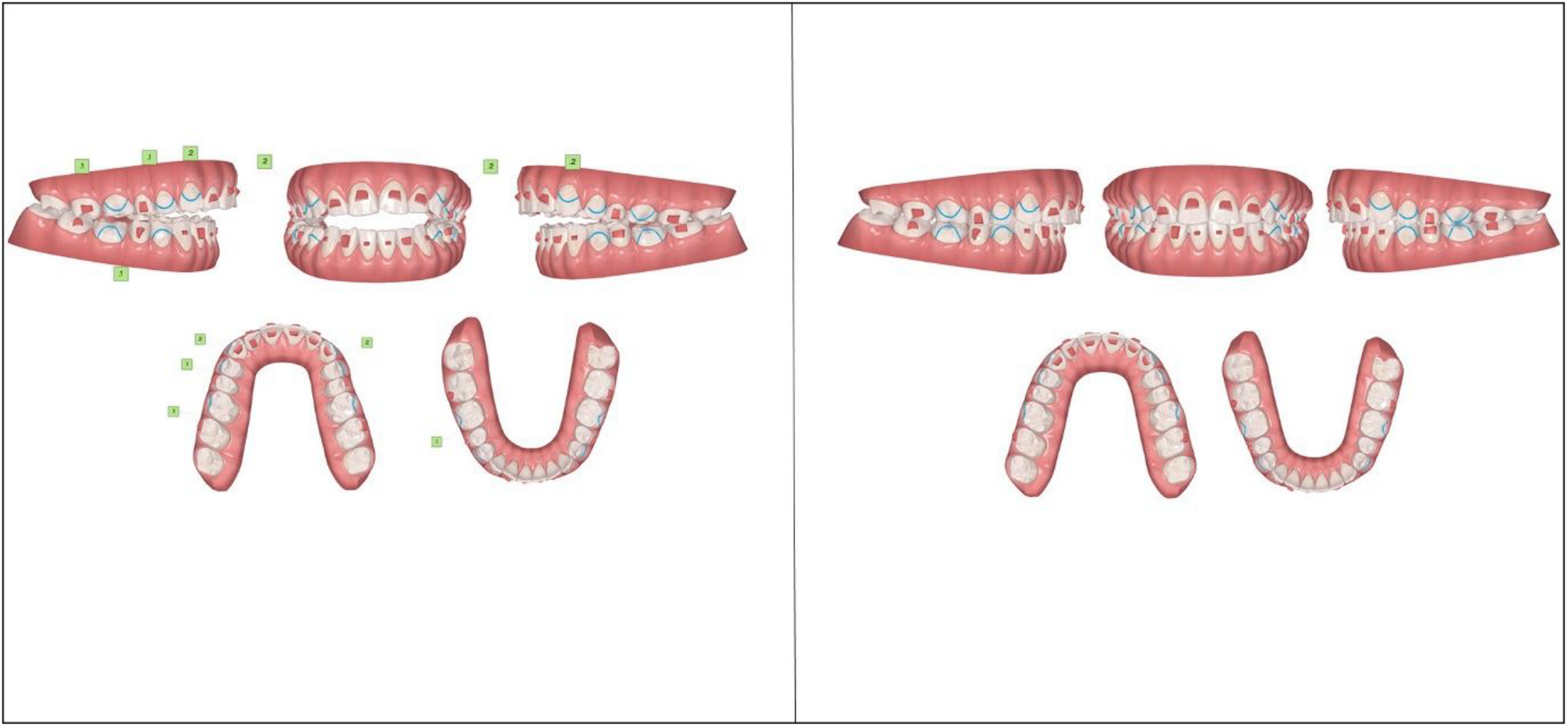
Pre‐ and post‐treatment ClinCheck™ assessment

On the third month of active treatment, two mini screws (1.8 × 8 mm) were placed at the infra zygomatic region (IZC) on both sides and a power chain (Force: 60 gms each) was applied to the lingual buttons placed on #15, #17, #25, and #27 for intrusion. (Figure 24).

**FIGURE 24:**
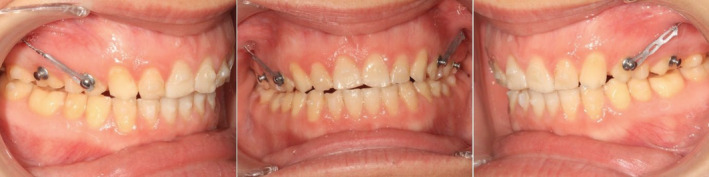
Mid‐treatment pictures showing intrusion of the posterior dentition using IZC mini implants

By the end of the 10th month of active intrusion, a good amount of counter‐clockwise movement of the mandible (autorotation) was generated reducing the overjet and the mandibular plane angle.(pre FMA: 28.83°; post FMA: 26.15°).

The AOB had closed, the increased overjet and posterior disocclusion had been addressed after 40 weeks of CAT. (Figure 25; Figure 26).

**FIGURE 25:**
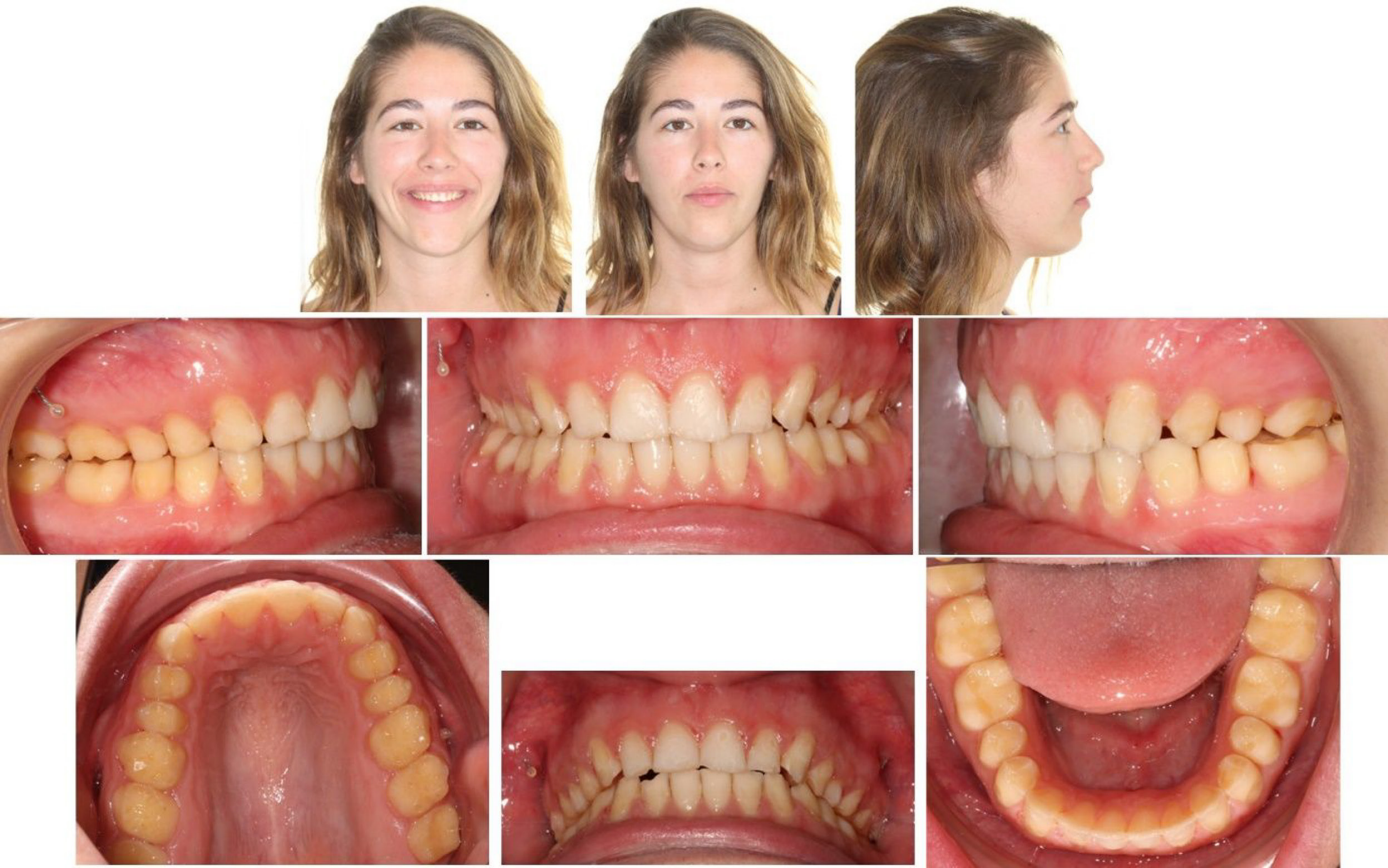
Post‐treatment intra‐oral and extra‐oral pictures

**FIGURE 26:**
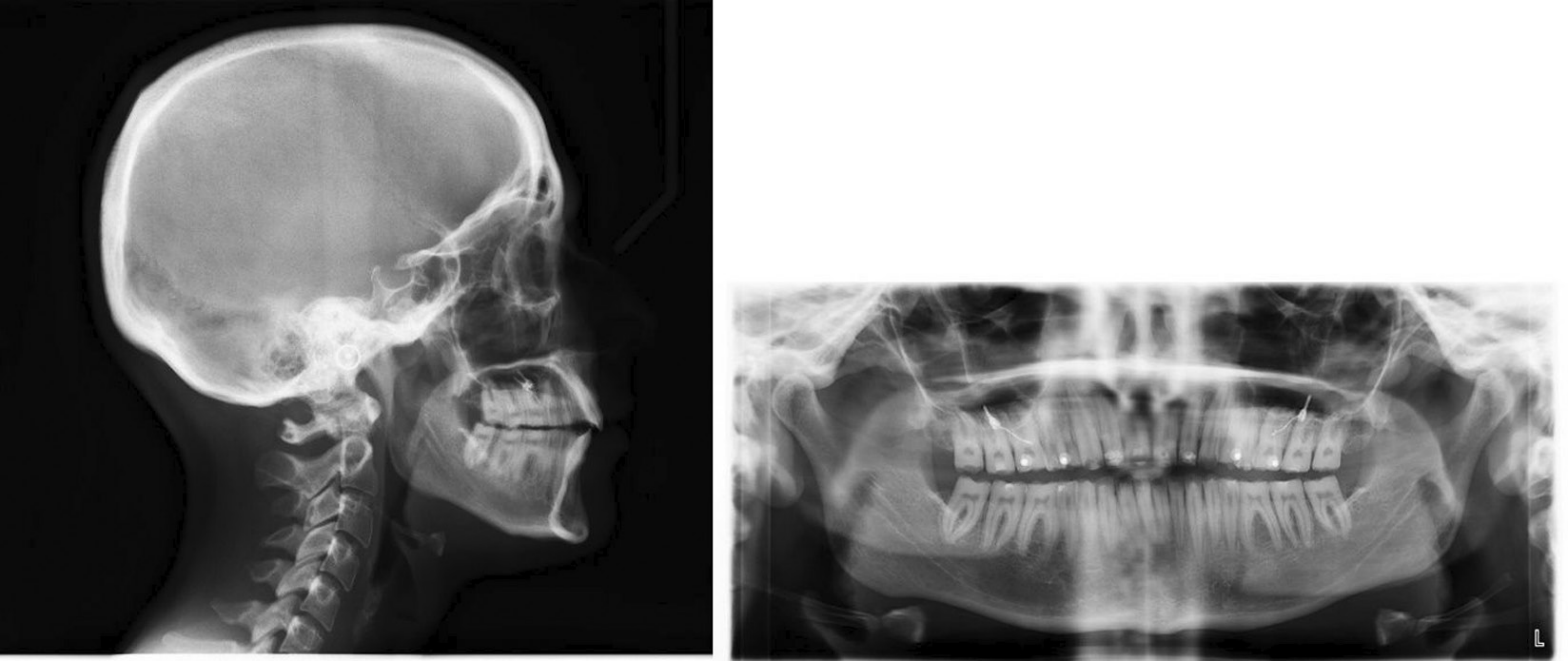
Post‐treatment radiographs

The final ClinCheckTM projections were nearly identical to the post‐treatment results; frontal superimpositions of the ClinCheckTM pre‐treatment analysis and post‐treatment projection revealed the amount of anterior extrusion and molar intrusion required to close the AOB. (Figure 27) Following the treatment, ViveraTM retainers were provided to retain the obtained results in both arches.

**FIGURE 27:**
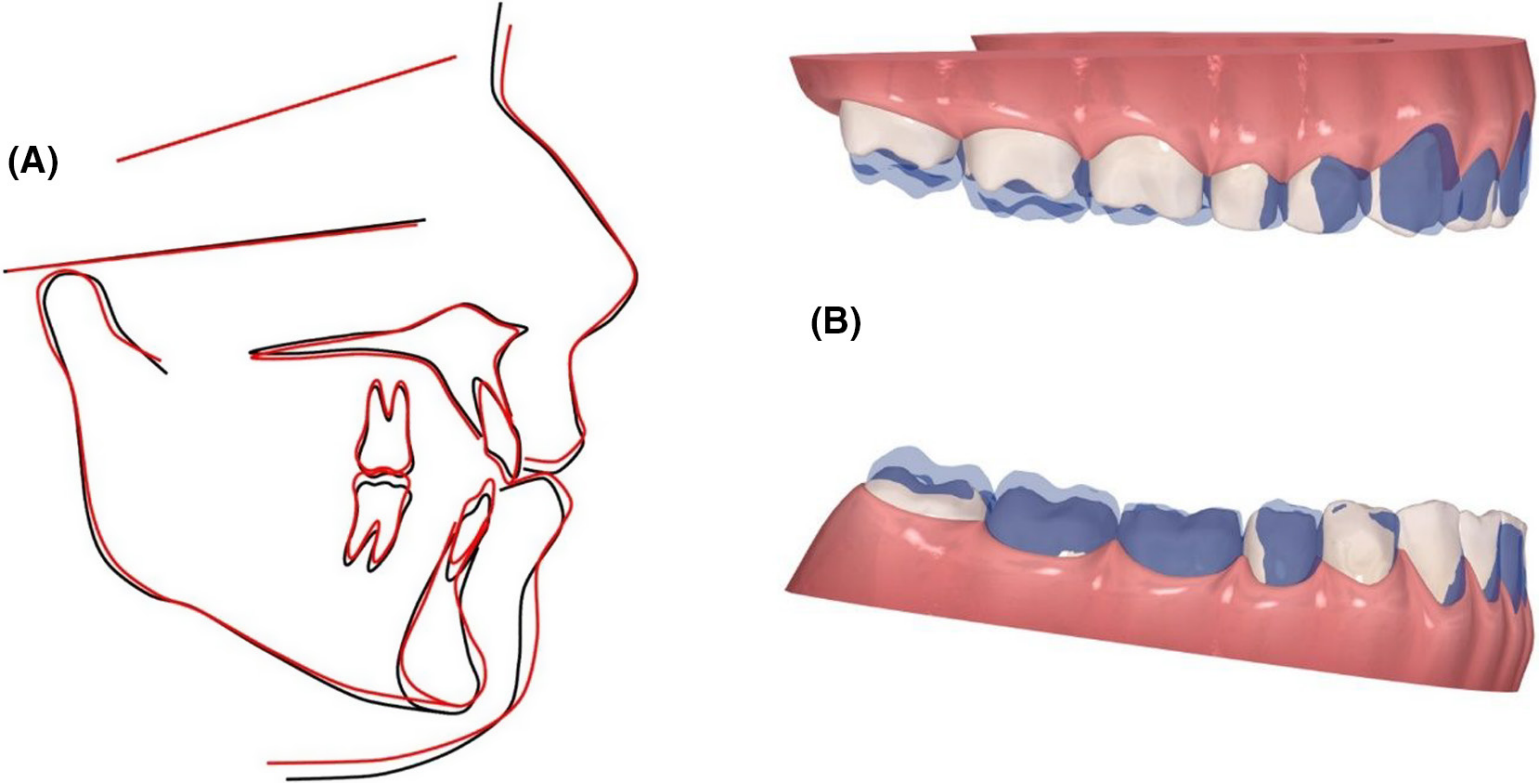
(A) Pre‐ and post‐treatment cephalometric tracings superimposed on the SN plane at S: showing intrusion of the upper molars and a counter clockwise rotation of the occlusal plane (B) Dental changes as seen in the ClinCheck™ software

## 5 DISCUSSION

Anterior extrusion, surgical impaction of the maxilla in adult patients, or molar eruption control in growing patients can all be used to address an AOB.^10^ There is currently no agreement on whether surgery or non‐surgical treatment is the most stable strategy for adult patients with AOB.^7^, ^11^ Several factors, particularly those AOB etiological factors, influence the related stability (or lack thereof). Tongue position and size, a persisting thumb sucking habit, occlusal determinants, respiratory problems, and/or adverse hereditary factors are just a few of them.^1^, ^12^

In correcting this type of malocclusion, aligners may be more effective than traditional braces, because they have less of an extrusive effect on the back teeth. Laura Talens‐Cogollos et.al,^13^ recently in a retrospective descriptive analytical study concluded that 74.2% of the subjects presented some degree of molar intrusion after CAT. Straight wire mechanics tend to have an extruding effect on the posterior teeth, which favors to aggravate the AOB.^14^, ^15^ Anecdotal evidence suggests that the covering plastic on the posterior teeth help to intrude the posterior teeth using the natural functional stomatognathic forces. Some also believe that a covering of anterior teeth with the aligners may aid in the restraining of habits such as tongue thrusting. Despite the lack of data to support these claims, several cases ranging from mild to severe AOB have been treated successfully with the mentioned benefits of CAT.^6^, ^11^

A relative open bite/dental open bite usually presents itself clinically by excessive incisor proclination.^16^ Among dental components, Sabri^17^ claimed that proclination of maxillary incisors can significantly reduce MIDR. This can be corrected by reducing incisor proclination, resulting in a relative extrusion of anterior teeth (drawbridge effect).^11^ Additional intermaxillary elastics or optimized attachments are not necessary for these maneuvers. According to the literature on anterior open bites treated using clear aligners, most of them suggest that the bite closure is achieved by a combination of maxillary and mandibular incisor extrusion along with maxillary and mandibular molar intrusion leading to a slight reduction in the mandibular plane angle.^18^, ^19^, ^20^, ^21^ (Table 4).

**TABLE 4:** Published literature on the dental changes following correction of anterior open bites using clear aligner therapy

Published literature	Study design	Sampling	Resulting tooth movement
Suh H et al.^18^	Retrospective	Sixty‐nine adult patients with an anterior open bite were recruited and divided into Angle's Classes I, II, and III. Fifty patients had skeletal open bite (mandibular plane angle [MPA] = 38°), while 19 had dental open bite.	The mean overbite change was 3.3 1.4 mm. Clear aligners alone resulted in 0.36 ± 0.58 mm of maxillary molar intrusion. The most important contributing parameters for open bite closure were maxillary incisor extrusion in patients with dental open bite and mandibular plane reduction with mandibular incisor extrusion in patients with skeletal open bite.
Harris K et al.^11^	Retrospective	The data analysis comprised 45 patients with a mean age of 30.73 ± 8.0 years and an initial open bite of −1.21 ± 1.21 mm.	The upper incisors extruded significantly (*p* 0.05) following treatment [U1‐SN'(mm) = 1.45 ± 0.85 mm]. L1‐MP'(mm) = 0.53 ± 0.74 indicates significant extrusion of the lower incisors. Upper and lower molar intrusion were statistically significant [U6‐SN'(mm) = −0.47 ± 0.59 mm; L6‐MP'(mm) = −0.39 ± 0.76 mm].
Garnett BS et al.^19^	Retrospective	There were two treatment groups of hyperdivergent adult patients (mandibular plane angles of ≥38°) with anterior open bites: 17 patients with fixed appliances and 36 patients with clear aligners.	Lower incisor extrusion was slightly greater in the clear aligner group (*p* = 0.009). Similar to both treatment groups, the basic mechanism of open bite correction was retroclination of the upper and lower incisors while maintaining the vertical position of the upper and lower molars.
Khosravi R et al.^20^	Retrospective	68 patients with normal overbites, 40 patients with deep bites, and 12 patients with open bites comprised the research sample. The median age of the patients was 33, and 70% of them were female.	Analyses of the patients with an open bite revealed a median deepening of 1.5 mm. The majority of improvements in open bite groups were attributable to changes in incisor position. The vertical position of the molars and the angle of the mandibular plane changed little.
Moshiri S et al.^21^	Prospective	Analyzed were the lateral cephalograms of 30 adult patients with anterior open bite who were treated with Invisalign (22 females, 8 males; mean age at initiation of treatment: 28 years and 10 months; mean anterior open bite at initiation of treatment: 1.8 mm).	The statistically significant mean increase in overbite was +3.4 mm; U1‐PP increased by 0.5 mm and U6‐PP decreased by 0.4 mm. However, these changes were not statistically significant. In contrast, L1‐MP increased by 0.8 mm while L6‐MP decreased by 0.6 mm. This suggests that mandibular molar intrusion and mandibular incisor extrusion are statistically significant in this sample.

Arch expansion and/or interproximal reduction can help gain space in both arches.

The arch shape, teeth size, and of course the periodontal condition all play a role in the screening of such cases.^12^ In case of a mild open bite (e.g. Case 1), it is feasible to get enough relative extrusion in order to fix the problem by CAT alone.^14^

The most demanding movements to replicate with aligners is clearly dental extrusion. Tooth extrusion in CAT is greatly influenced by the presence or absence of attachments. When pure extrusion of 0.5 mm or more is recognized, the software automatically places extrusive and anchorage‐optimized attachments.^15^ Conventional attachments (with a beveled edge toward the gingiva) allow for appropriate pressure from the aligner in order to extrude teeth. If aesthetics are a priority, these attachments might be placed on the palatal surface, too. In cases wherein greater aligner fit is required, the attachments may be placed both on the labial and palatal surfaces.^22^

Relative and absolute extrusion of the incisors are effectively controlled by using large rectangular‐shaped attachments with beveled edges toward the gingiva—placed as incisally as possible.^23^, ^24^ Use of additional intermaxillary elastics may aid with their extrusive movements in AOB cases of moderate severity (as seen in case 2).

Often a clockwise (downward) rotation of the maxilla is associated with an excessive lower anterior facial height (LAFH) going hand in hand with a hyperdivergent pattern, resulting in increased gingival show when smiling.^25^ The clinician's task is to avoid any posterior extrusion during leveling and alignment, as well as any anterior extrusion that might exacerbate a gummy smile.

While treating a severe AOB, vertical control of the posterior teeth is crucial. To resolve class II malocclusions with associated AOB, the biomechanical technique using IZC screws allows posterior intrusion generating a counterclockwise rotation of the jaw (shown in the case 3) resulting in a reduced mandibular plane angle and an increase in chin projection.^26^, ^27^

Simulated rotation of the mandible in the ClinCheck™ analysis can be helpful, if intrusion of the posterior segments is planned in such cases.

According to the literature, it is challenging to treat extrusion, rotation, or increased overjet with clear aligners. More specifically, a systematic review of the effectiveness of clear aligner therapy revealed that it is effective in controlling anterior intrusion but ineffective in controlling anterior extrusion; in controlling posterior buccolingual inclination but ineffective in controlling anterior buccolingual inclination; in controlling bodily movements of the upper molars of about 1.5 mm; and in controlling rotation of rounded teeth in particular.^28^ To get the best outcome possible, ”refinement” (further intervention during therapy) is frequently required. Other drawbacks include the greater expense of an Invisalign® treatment compared to fixed‐appliance therapy. Additionally, the orthodontist frequently needs more time to formulate a plan for clear‐aligner treatment than for fixed‐appliance therapy.^29^

## 6 CONCLUSION

A thorough diagnostic distinction is critical in selecting the right corrective treatments in open bite cases. Fixed appliances, either labial or lingual, are frequently used in non‐surgical open bite therapy. With the incorporation of extra‐radicular screws, it is possible now to plan more complex maneuvers orthodontically without the need for orthognathic surgery. Clear aligner therapy, on the contrary, has become increasingly popular in the treatment of complicated situations, including open bite malocclusions. The authors of this study report three distinct clinical situations in which open bite cases were successfully treated using clear aligners with and without adjuncts.

## AUTHOR CONTRIBUTIONS

Waddah Sabouni treated the case. Adith Venugopal and Samar M. Adel were involved in writing the case report. Waddah Sabouni and Nikhilesh Vaid were involved in supervising, diagnosing, and treatment planning of the case. Adith Venugopal proofread the final manuscript.

## FUNDING INFORMATION

No funding received for this study.

## CONFLICT OF INTEREST

The authors declare no conflict of interest.

## ETHICAL APPROVAL

This study did not require an ethical approval from any review board.

## CONSENT

The signed consent form is available with the principal author.

## Data Availability

The data used to support the findings of this study are included within the article.
